# Risk factors of Oral cancer- A hospital based case control study

**DOI:** 10.4317/jced.54618

**Published:** 2018-04-01

**Authors:** Natasha Azhar, Maheen Sohail, Fareeha Ahmad, Shaheen Fareeha, Soofia Jamil, Nouman Mughal, Hira Salam

**Affiliations:** 1 Dow International Dental College, Dow University Of Health Sciences; 2 Dow International Medical College, Dow University Of Health Sciences

## Abstract

**Background:**

Oral cancer is a highly prevalent malignancy in Pakistan. Among various risk factors associated with this neoplasm, habits such as smoked and smokeless tobacco usage, betel quid, and betel nut consumption are the major culprits in our society. In the present study, we aimed to ascertain prevalent risk factors for OC in our population and to compare our findings with healthy controls to establish their significance.

**Material and Methods:**

A hospital-based case control study was conducted at Dow University of Health Sciences, Pakistan from January 2015 – September 2016. Information pertaining to unhealthy oral habits was obtained from 62 oral cancer patients (cases) and 62 healthy controls on specifically designed proforma by the principal investigator.

**Results:**

Smokeless tobacco is strong, independent risk factor for oral cancer development in our study population. Buccal mucosa is the predominantly affected site (71%) which corresponds with high smokeless tobacco use. All studied habits increase risk of oral cancer as demonstrated by high odds ratio.

**Conclusions:**

Despite advancement in our knowledge and understanding of carcinogenic potential of these hazardous substances not enough efforts have been put forth to effectively control their widespread sale and consumption, particularly by the youth in our society.

** Key words:**Betel Quid, Gutka, Oral Cancer Risk, Smokeless Tobacco.

## Introduction

Head and neck cancers rank third in most common malignancies encountered in both genders globally. A subtype of head and neck cancers is oral cancer (OC), which is described as a cancerous growth in the mouth (1). Each year, over 450,000 patients are diagnosed with OC worldwide (2). In the last decade, researchers have observed an increase in incidence in younger patients, especially with cancers involving the tongue (3). OC has variable geographic distribution, being prevalent in Asian countries, particularly South and Southeast Asia (4).

OC embodies a plethora of malignances including, but not limited to squamous cell carcinoma (SCC), basal cell carcinoma (BCC), verrucous carcinoma, nasopharyngeal carcinoma (NPC), malignant melanoma, ameloblastoma, mucoepidermoid carcinoma etc. An astounding majority (~95%) of malignancies diagnosed in oral cavity are SCC originating in the mucosa lining mouth, tongue and lips whereby latter two are most commonly recorded sites (5-7).

Owing to grim deficiency in public awareness and scarcity of affordable screening tools, an alarming capacity of OC patients remain undetected until the disease has greatly advanced (8). Clinical presentation can be non-specific and exhibits substantial variation also hindering diagnosis. OC may present as skin lesions, mucosal ulcerations, a lump in the neck or anywhere in the oral cavity, and hyperpigmentation or depigmentation of mucosa. Three well-recognized presentations include a white patch (leukoplakia), a red patch (erythroplakia) or as a red and white patch (speckled leukoplakia) on oral mucous membrane, which is usually painless in its beginning stages (9). A burning sensation may be also be felt by the patient when lesion reaches an advanced stage along with dysphagia (9).

Pakistan is burdened with one of the highest incidence rates of OC in the world with strong male preponderance (10). Several risk factors have been established that contribute to observed prevalence trends in our population, including specific habits such as tobacco, alcohol, paan (betel quid), smokeless tobacco (including chewing tobacco and snuff), and betel nut consumption rendering population bearing lower socioeconomic status more susceptible to OC. Simultaneous consumption of some of these products, like tobacco and alcohol, is known to produces a synergistic effect on carcinogenesis (5). Besides habits, rising infection with certain viruses, like DNA viruses, Epstein Barr virus (EBV) and Human Papilloma virus (HPV) (especially HPV-16 and HPV-18) have also been reported to play a key role in OC pathogenesis (11). Importance of HPV in OC pathogenesis is highlighted by the fact that the 2017 update of 4th edition of World Health Organization (WHO) classification of head and neck tumors has recognized HPV-related SCC as a distinct entity (12).

In the present study, we aimed to ascertain prevalent risk factors for OC in our population and to compare our findings with healthy controls to establish their significance. We predicted that frequency of consumption would be directly proportional to OC incidence. We also expected participants consuming two or more of these products simultaneously to experience a synergistic effect.

## Material and Methods

A hospital-based case control study was conducted in the Dental outpatient department (OPD) at Dow International Medical and Dental College (DIDC) in Karachi, Pakistan from January 2015 until September 2016. Biopsy proven OC patients receiving treatment at Oral Surgery OPD at DIDC were enlisted in the study as cases, whereas healthy patients coming for routine dental checkup at Oral Diagnosis department during study period were recruited to participate in the study as controls. Informed consent was obtained before initiation of volunteering participants.

A case was defined as patients with histologically confirmed OC visiting DIDC during study period. The control group comprised of age-matched patients who visited the Department of oral diagnosis during the study period and did not have any significant medical history, including any malignancies or premalignant lesions. People with any other malignancy and comorbidities were excluded.

Sample size was calculated with the help of expert opinion. The sample size was calculated as 62 cases and 62 controls. Allocation ratio was kept as 1:1, hence for every case there was, one control was selected.

The investigator personally interviewed cases and controls by using a structured questionnaire. The questionnaire was validated using expert opinion and pre-tested prior to its use in the field. Informed consent was obtained from all participants before data collection. Medical ethics approval for this study was obtained from Institutional Review Board (IRB) of Dow University of Health Sciences (DUHS). The questionnaire included demographic information for the correlation of patients’ age, gender, medical history and date of diagnosis. The second part of the questionnaire included questions on oral hygiene practices and anticipated risk factors, from which we were able to conclude type, frequency, and duration of risk factors. The following products were grouped under ‘smokeless tobacco’ category: gutka, mawa, mainpuri, and naswar. The third part of questionnaire enabled exact characterization of patients’ oral condition that helped us in determining early signs, if any, related to OC, for instance; ulcer, red patch, white patch, mixed red-white patch, lump in the mouth or neck etc.

The data was analyzed using SPSS version 16. Univariate logistic regression was done followed by multivariate logistic regression for identifying the risk factors and adjusting for the confounding variables. Mann-Whitney U test was used for mean comparison between two study groups with the level of significance set as *p*<0.05.

## Results

A total of 124 participants were enrolled in study, 50% cases (n=62) and 50% controls (n=62). The participants’ demographics are summarized in  Table 1 and  Table 2.

**Table 1:**
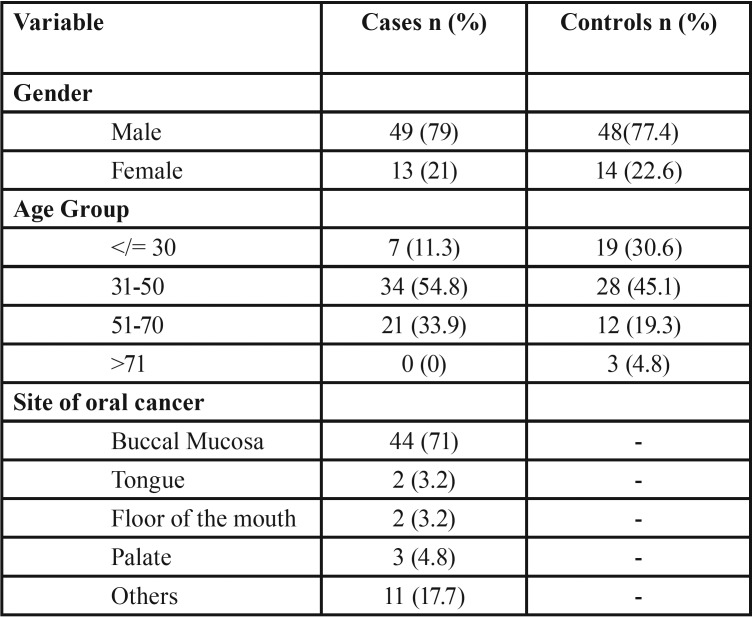
Demographic data of study participants: Age, gender, and site distribution of cases and controls.

**Table 2:**
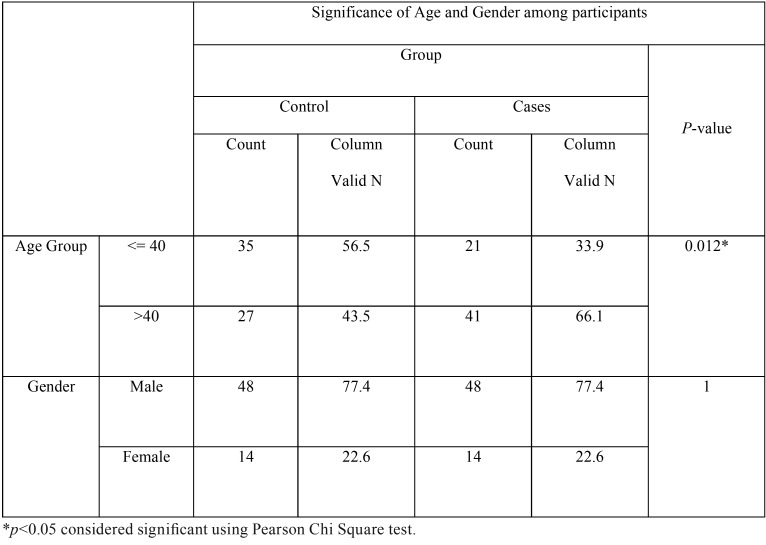
Statistical association with Age and Gender. A statistically signinficant difference exists between Cases and Control in Age Group but not in Gender.

The mean age of the respondents was 45 years with a standard deviation of 11 years for cases (Range: 27 – 70 years) and 40 years with a standard deviation of 16 years for controls (Range: 7 – 81 years). Majority of study participants were in the age group between 31 and 40 years.

Among the 62 cases, an overwhelming majority (71%) was recorded in buccal mucosa, making latter the most susceptible site in our study population ( Table 1).

A significant difference was observed in smokeless tobacco consumption between cases and controls, whereby 15% of study participants that reported habit of smokeless tobacco consumption, 63% were cases and 37% were controls. A slightly greater difference was recorded when participants consumed smokeless tobacco with other habit(s) (14.5% of participants), with 72% recorded in cases compared with 28% in controls ( Table 3).

**Table 3:**
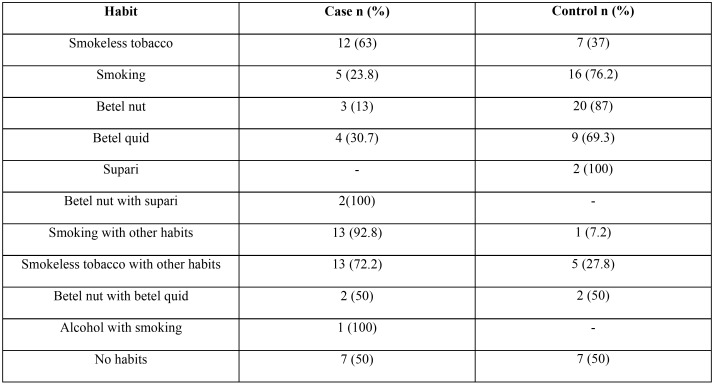
Distribution of habits among cases and controls. Among the cases, 42% of the patients consumed smokeless tobacco alone and in combination with other products. 8% smoked cigarettes and 17.7% smoked cigarettes in combination with other products, 12.9% from cases presented without habits.

An interesting trend was noted in smokers, those participants who smoked alone, without any other habit (17% of the respondents) only 23.8% were cases and 76.2% were controls. On the contrary, respondents who used cigarette with other habit(s) (11.3%) encompassed 92.8% cases and mere 7.2% of controls. Another interesting finding was that 11.3% of cases (n=7) reported no history of habitual use for any of the anticipated risk factors ( Table 3).

Using Odds ratio and Mann Whitney U test, we were able to conclude a significant association of smokeless tobacco consumption with head and neck squamous cell carcinoma incidence in our study population ( Table 4, Table 5).

**Table 4:**
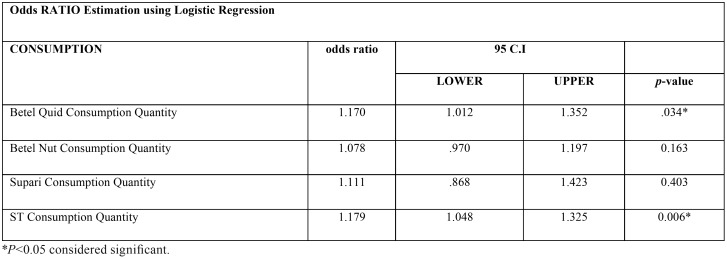
Association of quantity of consumption with oral cancer using Odds RATIO Estimation using Logistic Regression. Significant association recorded with smokeless tobacco (ST) consumption.

**Table 5:**
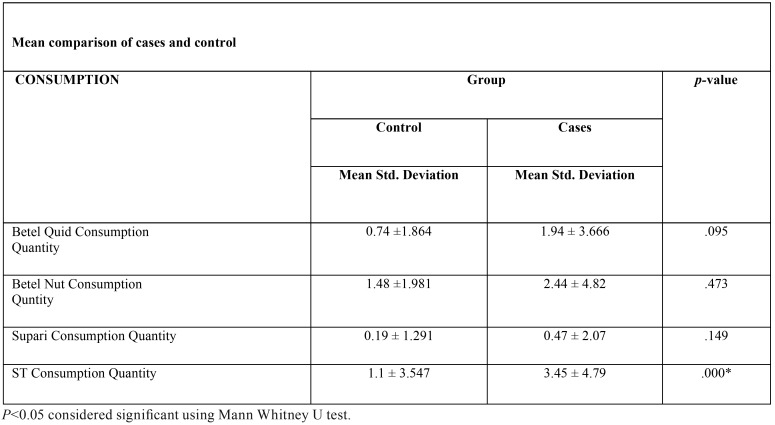
Association of quantity of consumption with oral cancer using Mean Comparison of cases and controls. Significant association recorded with smokeless tobacco (ST) consumption.

## Discussion

An 8 to 10-fold increase in oral cancer risk has been reported in Pakistan recently and consumption of tobacco-related products has been identified as the main culprit for this surge(13). The present study was undertaken to appraise risk of oral cancer development in relation to use of these products.

Exposure to pertinent risk factors plays a key role in determination of predominant tumour site in particular geographical region (14). Studies from India, Japan, Taiwan, Thailand, Yemen, and Iran have reported that tongue encompassed an estimated 42% of all oral cancer cases in these regions (14). Cancers involving labial mucosa predominate in Myanmar region (14). In Pakistan, however, oral cancer shows a strong predilection for involvement of buccal mucosa in both genders, a finding consistent with present study (15). The most frequently afflicted site is an indication for carcinogenic potential of habits such as smokeless tobacco use. Various smokeless tobacco products such as niswar and gutka are placed chronically in the buccal sulcus by users, a plausible explanation for observed association with buccal squamous cell carcinoma.

Consistent with previously reported studies, we found smokeless tobacco consumption to be a widely consumed and significant risk factor for oral squamous cell carcinoma (16-18). Despite increasing awareness among scientists of threat posed, tobacco-related products remain a popular health risk in Pakistan. Easy access to these carcinogenic substances by even school children and their addiction potential as well as affordability are major hindrances to curb this vice from our society (13).

In smokeless tobacco products, nitrosamines have been recognized as most potent carcinogens with their metabolites expressed in saliva and body fluids (19). A combined carcinogenic effect of smokeless tobacco has also been indicated with Herpes simplex virus 1 (HSV-1), and high-risk HPV but this association remains to be clearly elucidated since some studies have indicated an inversely proportional relationship between tobacco consumption and HPV DNA detection in tumour specimens warranting further research to characterize the association, if any (19).

In present study both, the duration and frequency of consumption of studied habits are increased in oral cancer patients. However, there is a greater emphasis on the effect that the period of consumption has on the patients. The maximum period of consumption was more than 10 years for cases and in controls it was 3 years. Consumption of smokeless tobacco combined with other products produced a synergistic effect that appeared to increase the risk of cancer, consistent with previous report(13). Betel nut was the most consumed risk factor probably because betel nut, also referred to as ‘chaalia’, is a very cultural product. Children tend to have easy access to it at because unlike cigarette smoking, it is not considered a taboo by Pakistani society and because it is extremely inexpensive.

## Conclusions

This study establishes a strong evidence for smokeless tobacco to be independent risk factor for oral cancer by showing significantly higher odds ratio of oral cancer development in these patients. Despite advancement in our knowledge and understanding of carcinogenic potential of these hazardous substances not enough efforts have been put forth to effectively control their widespread sale and consumption, particularly by the youth in our society.
